# Identification of Immunodominant Responses to the *Plasmodium falciparum* Antigens PfUIS3, PfLSA1 and PfLSAP2 in Multiple Strains of Mice

**DOI:** 10.1371/journal.pone.0144515

**Published:** 2015-12-11

**Authors:** Rhea J. Longley, Benedict R. Halbroth, Katie J. Ewer, Adrian V. S. Hill, Alexandra J. Spencer

**Affiliations:** The Jenner Institute, University of Oxford, Oxford, United Kingdom; University of Iowa, UNITED STATES

## Abstract

Malaria, caused by the *Plasmodium* parasite, remains a serious global public health concern. A vaccine could have a substantial impact on eliminating this disease, alongside other preventative measures. We recently described the development of three novel, viral vectored vaccines expressing either of the antigens PfUIS3, PfLSA1 and PfLSAP2. Each vaccination regimen provided high levels of protection against chimeric parasite challenge in a mouse model, largely dependent on CD8^+^ T cells. In this study we aimed to further characterize the induced cellular immune response to these vaccines. We utilized both the IFNγ enzyme-linked immunosorbent spot assay and intracellular cytokine staining to achieve this aim. We identified immunodominant peptide responses for CD4^+^ and CD8^+^ T cells for each of the antigens in BALB/c, C57BL/6 and HLA-A2 transgenic mice, creating a useful tool for researchers for subsequent study of these antigens. We also compared these immunodominant peptides with those generated from epitope prediction software, and found that only a small proportion of the large number of epitopes predicted by the software were identifiable experimentally. Furthermore, we characterized the polyfunctionality of the induced CD8^+^ T cell responses. These findings contribute to our understanding of the immunological mechanisms underlying these protective vaccines, and provide a useful basis for the assessment of these and related vaccines as clinical constructs.

## Introduction

Malaria, caused by the *Plasmodium* parasite, remains an infectious disease of global concern and there is widespread agreement that a vaccine is needed to eliminate this pathogen [1]. Whilst recent results using the pre-erythrocytic sub-unit vaccine RTS,S/AS01 are encouraging [2], substantial increases in efficacy and durability are still required.

Research in our laboratory has focused on a viral vectored, prime-boost sub-unit vaccination approach [3], and we recently demonstrated success using the *P*. *falciparum* pre-erythrocytic antigens liver-stage antigen 1 (PfLSA1), liver-stage associated protein 2 (PfLSAP2) and up-regulated in sporozoites 3 (PfUIS3) [4]. We demonstrated that both PfLSA1 and PfLSAP2, when delivered using the viral vectors chimpanzee adenovirus 63 (ChAd63) and modified vaccinia virus Ankara (MVA) with an eight-week interval, could protect 70–87.5% of both inbred and outbred mice against chimeric *P*. *berghei* parasites expressing the cognate *P*. *falciparum* antigen. Whilst PfUIS3 did not deliver such high levels of sterile efficacy when delivered in the same viral vectors, it provided a significant delay in the time to patent parasitaemia, equal to that of *P*. *falciparum* circumsporozoite protein (CSP) (the antigen targeted by RTS,S vaccination).

We further determined that the presence of CD8^+^ T cells was crucial for protection [4]; the induction of exceptionally high CD8^+^ T cell responses is a key feature of this prime-boost approach [5–7]. It has long been known that cellular responses against the liver-stage are essential for protection induced by irradiated sporozoite vaccines [8–11], arguably the most successful vaccination regimen against *P*. *falciparum* developed so far. However, the actual mechanism by which these CD8^+^ T cells provide protection is still largely unknown [12].

In this study we therefore aimed to further investigate the cellular immunological response induced by these vaccines. We sought to identify the immunodominant peptide responses in BALB/c and C57BL/6 mice to allow future studies of the specific T cells involved in protection and to allow the design of epitope-based vaccines. A model was also available to assess the presence of HLA-A2-restricted responses within these antigens: transgenic mice expressing human leukocyte antigen A2 (HLA-A2) [13]. HLA-A2 is a common major histocompatibility complex type in the general human population [14], and hence finding an HLA-A2-restricted response would suggest a high likelihood of immunogenicity in humans and facilitate immune-monitoring in clinical trials of vaccines expressing these antigens. Furthermore, we wanted to assess the polyfunctionality of the induced immune response, by assessing the populations of cytokines secreted from antigen-specific T cells: the polyfunctionality, or strictly monofunctionality, of CD8^+^ T cell responses have been correlated with vectored vaccine efficacy in controlled human malaria infection phase II efficacy trials [15].

We present in this study immunodominant peptide responses to these antigens in mice, and data showing most cells induced were polyfunctional, producing both interferon-gamma (IFNγ) and tumor necrosis factor-alpha (TNFα). These findings contribute not only to our understanding of the immunological mechanisms of these newly developed vaccines, but also provide a useful tool for subsequent research in the form of identified immunodominant regions.

## Materials and Methods

### Animals

Female BALB/c and C57BL/6 mice, of at least six weeks of age, were purchased from Harlan, UK. A breeding pair of HLA-A2 transgenic (tg) mice [13] was kindly provided by Vincenzo Cerundolo (Weatherall Institute of Molecular Medicine, Oxford) and this strain was then bred in-house.

### Ethics statement

All animal work was conducted in accordance with the UK Animals (Scientific Procedures) Act 1986 and approved by the University of Oxford Animal Care and Ethical Review Committee for use under Project License PPL 30/2414 or 30/2889. Animals were group housed in individually ventilated cages under specific pathogen free conditions, with constant temperature, humidity and with a 12:12 light-dark cycle (8am to 8pm). For induction of short-term anaesthesia, animals were anaesthetized using vaporized IsoFlo. All animals were humanely sacrificed at the end of each experiment by an approved Schedule 1 method (cervical dislocation). All efforts were made to minimize suffering.

### Genotyping of HLA-A2 transgenic mice

To confirm expression of the human HLA-A2 gene, DNA was extracted from ear punch biopsies by incubation in 50mM Tris pH 8, 2mM NaCl, 10mM EDTA, 1% SDS and 1mg/ml proteinase K in dH_2_O for 40 minutes at 55°C prior to heat inactivation of proteinase K at 99°C for five minutes. PCR was performed using Reddymix PCR Mastermix (Thermo Fisher Scientific, USA), according to the manufacturer’s instructions. Primers were designed using the online software program Primer3 [16] for HLA-A2, H-2D and human and mouse beta-2 microglobulin (β2m) (S1 Table). Control DNA was collected from the HepG2 cell line (HLA-A2) and C57BL/6 mice (H-2D^b^). Genotyping results indicated expression of HLA-A2 and mouse β2m and the ability to mount a HLA-A2 restricted response was confirmed by vaccination of mice with MVA NP+M1 [17] and detection of a strong response to an Influenza A HLA-A2-restricted epitope [18, 19] (S1 Fig).

### Vaccines, immunizations and antigens used for *in vitro* restimulation

The generation of the ChAd63 and MVA vectored vaccines containing either of the inserts PfUIS3, PfLSA1 and PfLSAP2 has previously been described in detail [4]. Mice were immunized intramuscularly (i.m.) into the musculus tibialis with a total volume of 50μl vaccine administered in endotoxin free D-PBS, with doses stated in the relevant figure legends. To measure the immune response in the various cellular immunoassays described below, cells were restimulated *in vitro* with a single peptide pool to the appropriate *P*. *falciparum* 3D7 antigen encompassing synthetic crude 20mers overlapping by ten amino acids (peptides synthesized by Neo Group Inc., USA, or Thermo Fisher Scientific). In the epitope mapping experiments single 20mer peptides were used. All peptide sequences are provided (see S2–S4 Tables).

### Spleen *ex vivo* IFNγ enzyme-linked immunosorbent spot (ELISpot) assay

Splenocytes were treated with ammonium-chloride-potassium (ACK) lysis buffer followed by stimulation for 18–20 hours with a final concentration of 1μg/ml of the appropriate peptide pool, or single peptide, in MAIP ELISpot plates (Mabtech, Sweden). IFNγ ELISpots were performed as previously described [20] using coating and detecting antibodies from MabTech. Spots were enumerated using an ELISpot plate counter (AID, Germany) and expressed as the number of spot forming units (SFU) per million splenocytes, after background subtraction from wells containing media and no peptide.

### Intracellular cytokine staining (ICS)

For intracellular cytokine staining, splenocytes were prepared as above or blood was lysed with ACK lysis buffer to isolate the peripheral blood mononuclear cells, followed by stimulation for six hours with a final concentration of 5μg/ml of the appropriate peptide pool, 1μg/ml Brefeldin A (BD Biosciences, UK) and anti-mouse CD107a-PE (clone 1D4B, eBioscience, UK). Cells were subsequently surface stained with anti-mouse CD16/32 (Fc block, clone 93, BD Biosciences), anti-mouse CD4-eFluor® 450 (clone RM4-5, eBioscience) and anti-mouse CD8α-PerCPCy5.5 (clone 53–6.7, BD Bioscience) followed by fixation with 10% neutral buffered formalin solution containing 4% paraformaldehyde (Sigma Aldrich, UK). Staining of intracellular cytokines was achieved using anti-mouse TNFα-FITC (clone MP6-XT22, BD Biosciences), anti-mouse interleukin 2 (IL-2)-PeCy7 (clone JES6-5H4, BD Biosciences) and anti-mouse IFNγ-APC (clone XMG1.2, eBioscience) diluted in Perm/Wash buffer (BD Biosciences). Data were acquired using a LSRII flow cytometer (BD Biosciences) and analysed using FlowJo (Tree Star Inc.).

### Polyfunctionality analysis

Polyfunctionality of T cells was analysed using the Boolean gate platform in FlowJo followed by subsequent preparation of data in Pestle (Mario Roederer, National Institutes of Health) for final analysis and graphical representation in SPICE (simplified presentation of incredibly complex evaluations, Mario Roederer [21]).

### Predicted epitopes

T cell epitopes within the three antigen sequences were predicted using two different servers: SYFPEITHI and the immune epitope database (IEDB). The strong H-2^d^-restricted epitope Pb9 from *P*. *berghei* CSP [22] was used as a comparison for epitope strength. Using SYFPEITHI, the higher the score the greater the likelihood the peptide is processed and presented, based on binding motifs [23]. Pb9 was given a score of 32. IEDB employs a consensus approach (combining ANN [24, 25], SMM [26] and CombLib [27]) to determine the likely ability of the sequence to bind MHC Class I molecules and the score is given as a percentile rank [28]. A small percentile rank indicates high affinity; Pb9 was given a percentile rank of 0.1. For predicted CD4^+^ epitopes IEDB also uses a consensus approach to combine different methods and the score is again given as a percentile rank, comparing the peptides average score of four methods against 5 million random 15mers selected from the SWISSPROT database [29, 30]. These predictions were made on the 23^rd^ and 25^th^ March 2015.

### Statistical analysis

The statistical software Prism version 5 (Graphpad, USA) was used for all analyses. Non-parametric data are shown as the median with individual data points plotted, unless otherwise indicated. A p value of less than 0.05 was considered significant.

## Results and Discussion

### Immunodominant responses to PfUIS3, PfLSA1 and PfLSAP2

For each antigen, immunodominant peptides were first identified by IFNγ ELISpot using splenocytes from mice vaccinated with the ChAd63-MVA regimen, as described in the Materials and Methods. To determine whether the identified immunodominant peptides elicited primarily CD4 or CD8 responses, mice were vaccinated with ChAd63 only and responses in the spleen measured by ICS.

**Fig 1:**
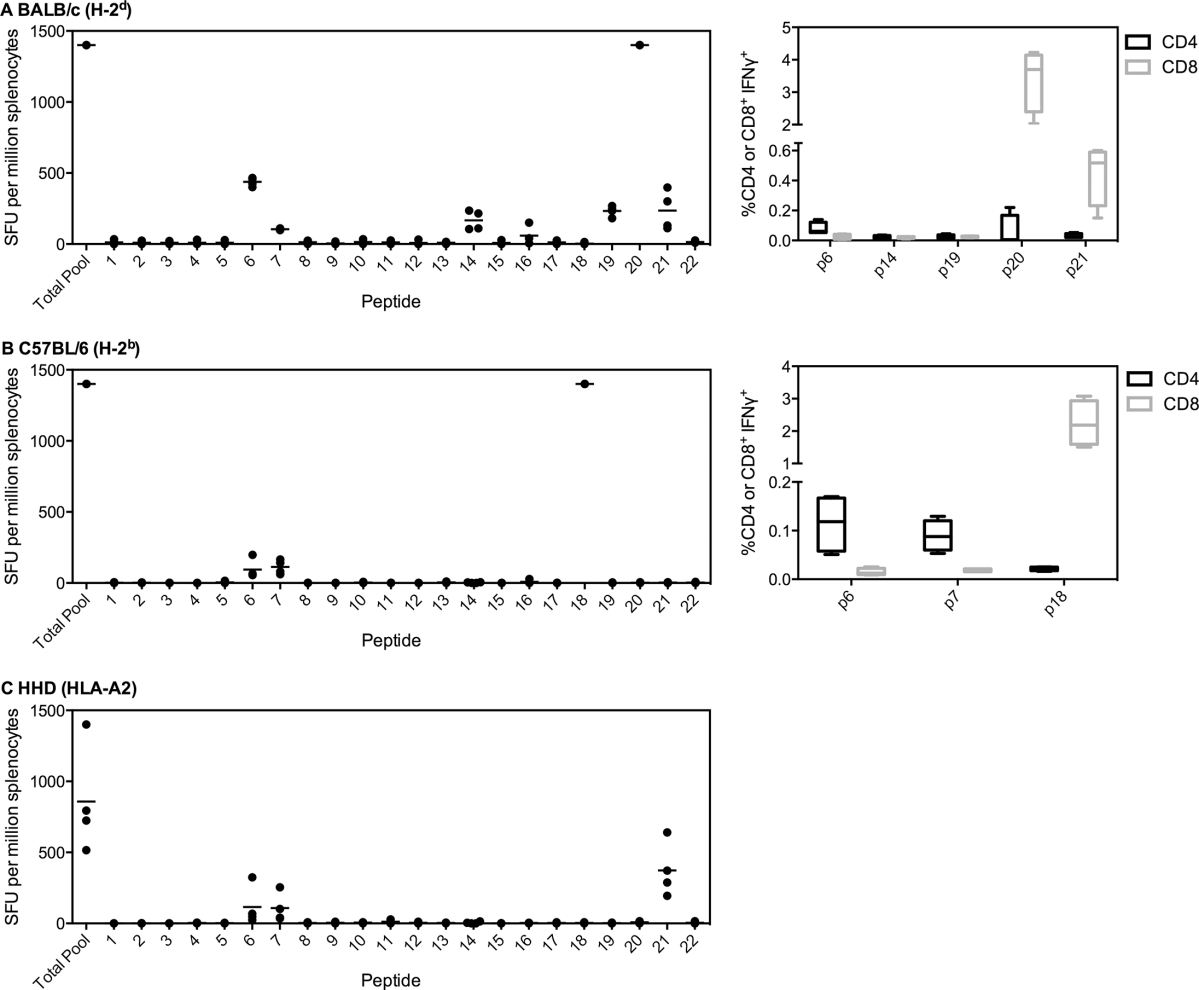
Immunodominant responses to PfUIS3. (A) BALB/c, (B) C57BL/6 or (C) HLA-A2 tg mice (n = 4 per strain) were vaccinated i.m. with 1x10^8^ infectious units (ifu) ChAd63-PfUIS3 followed eight weeks later by 1x10^6^ plaque forming units (pfu) MVA-PfUIS3. Two weeks post-MVA boost, mice were sacrificed and splenocytes isolated to perform an *ex vivo* IFNγ ELISpot. Splenocytes were stimulated with either an overlapping peptide pool to PfUIS3 or individual peptides (20aa each, overlapping by ten). Both median and individual data points are shown. For (A) BALB/c and (B) C57BL/6, CD4^+^ and CD8^+^ epitopes were also determined (right panel). Two weeks post-ChAd63 (n = 4 per strain), splenocytes were isolated and incubated with the appropriate peptide for six hours prior to ICS staining. Box plots show the percentage IFNγ^+^ of CD4^+^ or CD8^+^ cells, with whiskers representing the maximum and minimum.

For PfUIS3, two peptides were identified that could be broadly recognized, as positive responses were seen in each strain of mice, representing amino acids (aa) 51 to 80 (labeled as number 6 and 7, Fig 1). The immunodominant response in BALB/c was elicited by peptide 20, aa191-210, along with five sub-dominant responses (including peptides 6 and 7). For BALB/c, peptides 20 and 21 elicited strong CD8^+^ responses, whereas peptide 6 elicited a weaker CD4^+^ response (Fig 1A). The immunodominant response in C57BL/6 mice was to peptide 18, aa171 to 190, and in HLA-A2 tg mice it was to peptide 21, aa201 to 220. For C57BL/6, peptides 6 and 7 elicited CD4^+^ responses, whilst the immunodominant peptide 18 was CD8^+^ (Fig 1B). This appears to be the first report of identification of immunodominant regions of PfUIS3. This finding warrants further investigation to determine whether the conserved responsive region contains a single epitope, and whether this epitope is, or epitopes are, protective, given protection was dependent on a cellular response [4].

**Fig 2:**
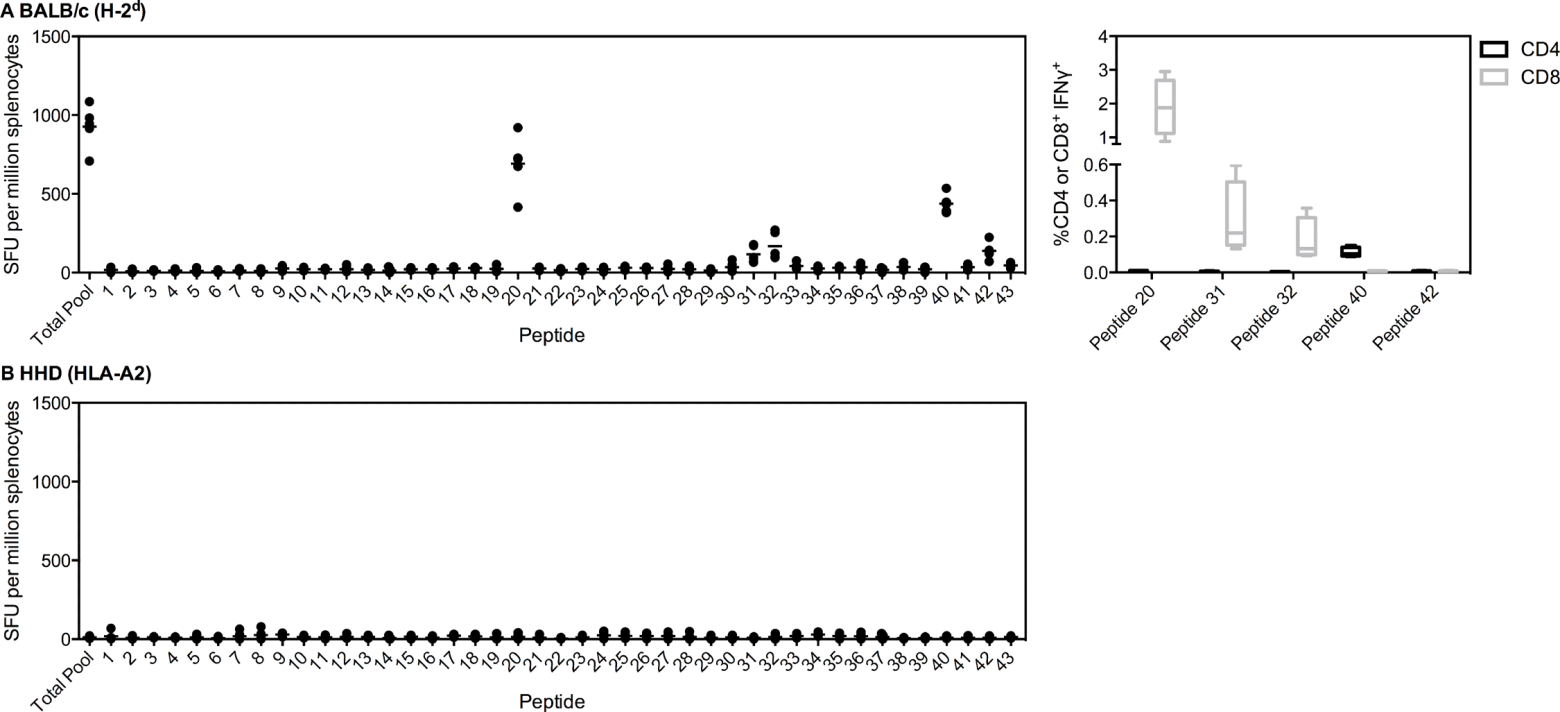
Immunodominant responses to PfLSA1. (A) BALB/c or (B) HLA-A2 tg mice (n = 5) were vaccinated i.m. with 1x10^8^ ifu ChAd63-PfLSA1 followed eight weeks later by 1x10^7^ pfu MVA-PfLSA1. Two weeks post-MVA boost, mice were sacrificed and splenocytes isolated to perform an *ex vivo* IFNγ ELISpot. Splenocytes were stimulated with either an overlapping peptide pool to PfLSA1 or individual peptides (20aa each, overlapping by ten). Both median and individual data points are shown. For (A) BALB/c, CD4^+^ and CD8^+^ epitopes were also determined (right panel). BALB/c mice (n = 4) were vaccinated with 1x10^8^ ifu ChAd63-PfLSA1 and two weeks later sacrificed and splenocytes isolated. Cells were incubated for six hours with the relevant peptide prior to ICS staining. Box plots show the percentage IFNγ^+^ of CD4^+^ or CD8^+^ cells, with whiskers representing the maximum and minimum.

For PfLSA1, immunodominant responses were only mapped in BALB/c and HLA-A2 tg mice as PfLSA1 is not immunogenic in C57BL/6 [4, 31, 32]. Immunodominant responses in BALB/c mice were identified to peptides 20 (aa918 to 937) and 40 (aa1118 to 1137), with three further subdominant responses (Fig 2A). Using ICS staining, peptides 20, 31 and 32 were found to elicit CD8^+^ responses whilst peptide 40 was shown to be CD4^+^ (Fig 2A). A version of PfLSA1 has previously been mapped; this vaccine is a 456aa polypeptide containing the N and C terminal regions, and two slightly differing copies of the 17aa repeats, the entirety known as FMP011 [31]. The immunodominant regions mapped were essentially the same as those currently identified. Our peptide 40 contains the sequence EKFIKSLFH, which Brando and colleagues identified to be the most immunogenic by IFNγ ELISpot. Brando and colleagues did not identify the other major responsive region we identified, as this region (aa918 to 937) was not included in their vaccine construct. This was the most immunogenic region identified in our study, and importantly elicited a CD8^+^ response. The lack of clinical efficacy of the protein in adjuvant vaccine studied by Brando and colleagues likely related to the lack of CD8^+^ T cell induction in their clinical trial [33]. The other epitopes identified in FMP011 constituted minor responsive regions in our vaccine construct (peptides 42 and 38). ChAd63-MVA PfLSA1 was found to be non-immunogenic in HLA-A2 tg mice (Fig 2B).

**Fig 3:**
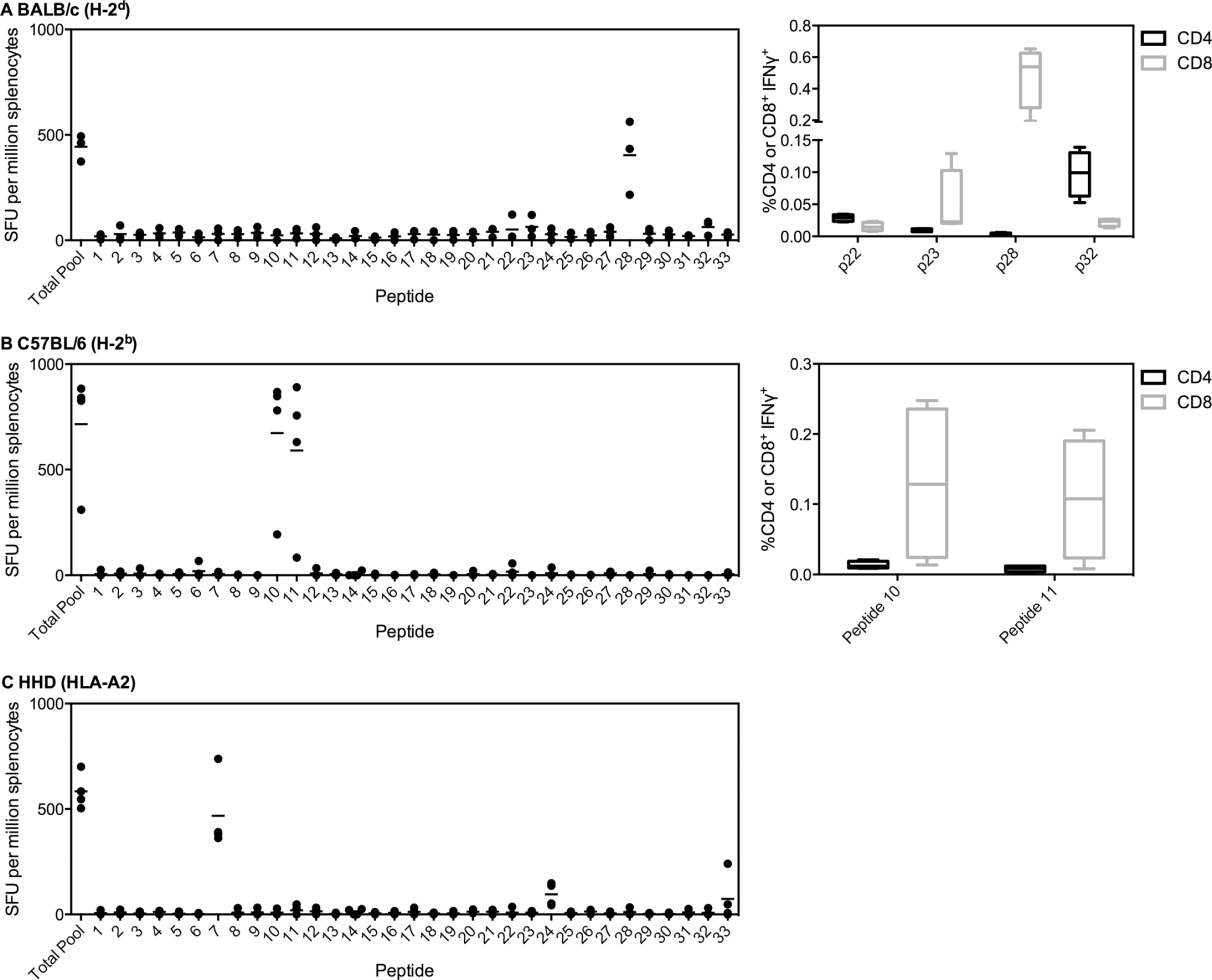
Immunodominant responses to PfLSAP2. (A) BALB/c (n = 3), (B) C57BL/6 (n = 4) or (C) HLA-A2 (n = 4) mice were vaccinated i.m. with 1x10^8^ ifu ChAd63-PfLSAP2 followed eight weeks later by 1x10^7^ pfu MVA-PfLSAP2. Two weeks post-MVA boost, mice were sacrificed and splenocytes were isolated to perform an *ex vivo* IFNγ ELISpot. Splenocytes were stimulated either with an overlapping peptide pool to PfLSAP2 or individual peptides (20aa each, overlapping by ten) covering the entire sequence. Both median and individual data points are shown. For (A) BALB/c and (B) C57BL/6, CD4^+^ and CD8^+^ epitopes were also determined (right panel). Two weeks post-ChAd63 (n = 4 per strain), splenocytes were isolated and incubated with the appropriate peptide for six hours prior to ICS staining. Box plots show the percentage IFNγ^+^ of CD4^+^ or CD8^+^ cells, with whiskers representing the maximum and minimum.

PfLSAP2 is a relatively recently identified liver-stage antigen [34], and our previous work constituted the first assessment of this antigen as a vaccine candidate [4]. Hence, this is the first time this antigen has been mapped for immunogenic regions. We identified one immunodominant response in BALB/c mice to peptide 28, aa241 to 260 (Fig 3A). ICS staining this peptide showed it to be MHCI-restricted (CD8^+^ T cell response). Of the minor immunodominant regions, peptide 23 was also CD8^+^ whereas peptides 22 and 32 were CD4^+^. Two immunodominant peptides were identified in C57BL/6 mice, peptides 10 and 11, covering aa61 to 90 (Fig 3B). Both peptides were MHCI-restricted. An immune response was also induced in HLA-A2 tg mice, with the immunodominant response to peptide 7, aa31 to 50 (Fig 3C).

For each of the three vaccines, the identified immunodominant responses represent not only a potential mechanism of protection, but also provide a new resource for others assessing the immune response to these antigens. The immunodominant peptide sequences and MHCI/MHCII-restriction are listed in Table 1. Whilst we performed epitope mapping using overlapping peptides of 20aa in length, the optimal length for class I binding peptides is generally accepted to be 8-10aa and for class II 12-24aa. Therefore to identify the minimal epitopes and to determine the true magnitude of the induced response [35], further high-resolution mapping will be required.

**Table 1:** Top immunodominant peptides and MHCI/MHCII-restriction.

Antigen	Strain	Peptide	Sequence	CD4^+^ or CD8^+^
PfUIS3	BALB/c	p6	AIEEHNKRKKLIYYSLIASG	CD4
		p20	GLQENRNISLSKYQENKAVM	CD8
		p21	SKYQENKAVMDLKYHLQKVY	CD8
	C57BL/6	p6	AIEEHNKRKKLIYYSLIASG	CD4
		p7	LIYYSLIASGAIASVAAILG	CD4
		p18	SNDQKDSHVNNMEYMQKFVQ	CD8
PfLSA1	BALB/c	p20	SENERGYYIPHQSSLPQDNR	CD8
		p31	EEEDDEDLDEFKPIVQYDNF	CD8
		p32	FKPIVQYDNFQDEENIGIYK	CD8
		p40	KNDKQVNKEKEKFIKSLFHI	CD4
PfLSAP2	BALB/c	p23	WHYSHSLLRDKFNKMKSSLW	CD8
		p28	ELLIKEHDDYNSIYNYINNE	CD8
		p32	FTMETFIKCKISLENNMRNV	CD4
	C57BL/6	p10	LIQNILLSNVSLISGSHLYK	CD8
		p11	SLISGSHLYKRNSRKFAEGY	CD8

### Comparison to epitope prediction software

We next compared the dominant immunogenic peptides identified through our ELISpot experiments to those predicted using two common epitope prediction servers, SYFPEITHI and IEDB (Table 2). Only two of the seven peptides (peptide 18 from PfUIS3 for H-2^b^, and peptide 20 from PfLSA1 for H-2^d^) received a high score from both prediction servers. The remaining dominant peptides were not predicted with high strength. Furthermore, of the top 3 epitopes predicted for each antigen at each MHC haplotype (H-2^d^, H-2^b^ and HLA-A2) by the servers, only a limited number were found to be immunogenic peptides *in vivo* (26% for SYFPEITHI, 19% for IEDB MHC Class I and 50% for IEDB MHC Class II) (S5–S7 Tables). Whilst the limited ability of the prediction server to identify our dominant peptides and choose immunogenic targets is perhaps not surprising [36], we only studied three antigens and used just two servers. Incorporating more predictions to create a more stringent definition of a ‘predicted epitope’ could result in greater success (reviewed in [37]), but our results do show the benefit of full experimental peptide mapping to clearly define immunodominant regions.

**Table 2:** Immunodominant regions and the accuracy of epitope prediction servers.

Antigen	MHC	Dominant peptide sequence^a^	SYF.^b^	IEDB (I)^c^	IEDB (II)^d^
PfUIS3	H-2^d^	GLQENRNISLSKYQENKAVM	<20	13	11.23
	H-2^b^	SNDQKDSHVNNMEYMQKFVQ	28	0.2	>50
	HLA-A2	SKYQENKAVMDLKYHLQKVY	23	3	N/A
PfLSA1	H-2^d^	SENERGYYIPHQSSLPQDNR	27	0.3	40.49
PfLSAP2	H-2^d^	ELLIKEHDDYNSIYNYINNE	23	0.7	73.06
	H-2^b^	LIQNILLSNVSLISGSHLYKRNSRKFAEGY	25	0.4	27.70
	HLA-A2	KKEKIKCGTFFGYIFLSKFM	<20	3.45	N/A

^a^ Dominant peptide identified in the ELISpot mapping assay.

^b^ Score given by SYFPEITHI prediction server.

^c^ Percentile rank given by IEDB MHC Class I binding server.

^d^ Percentile rank given by IEDB MHC Class II binding server.

### Polyfunctionality of the immune response

**Fig 4:**
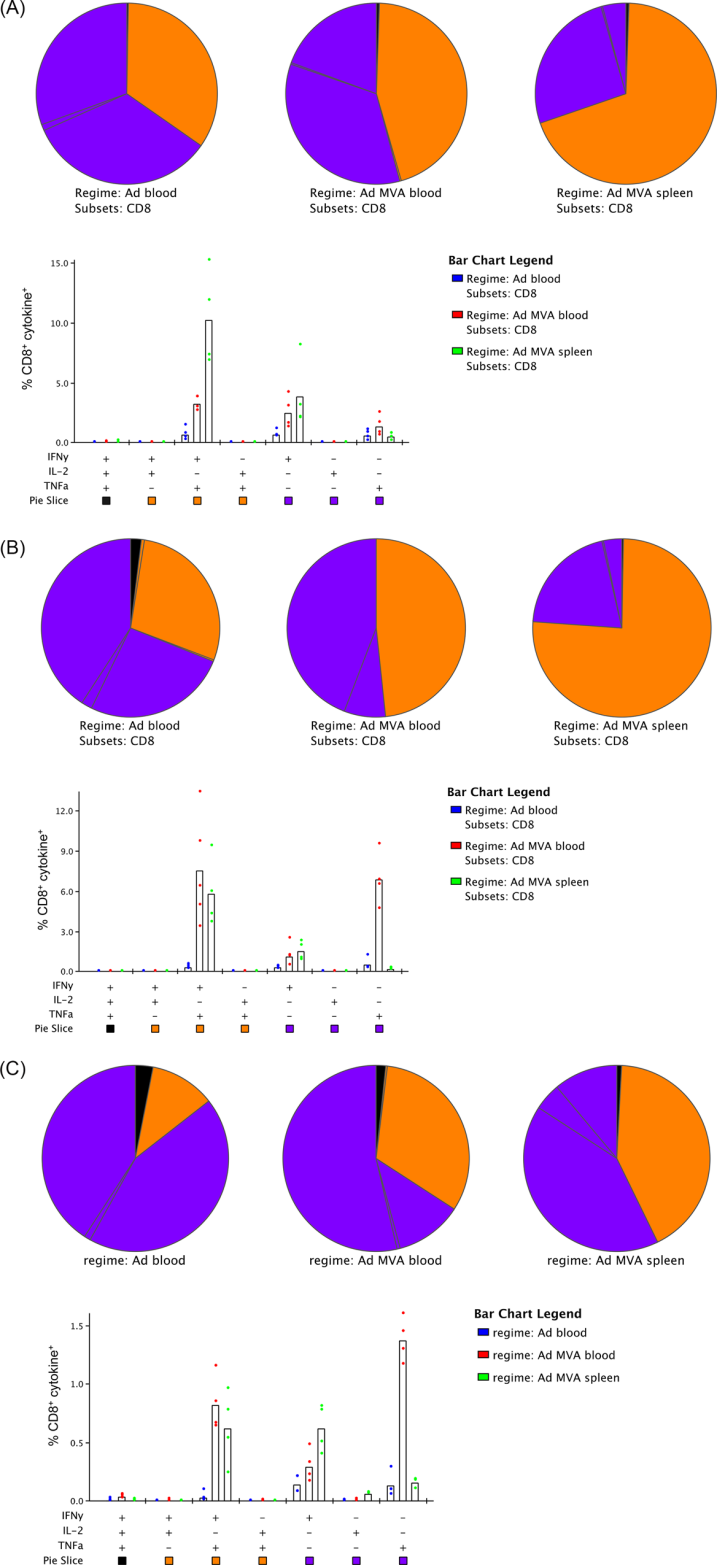
Polyfunctionality of CD8^+^ T cells induced by ChAd63-MVA vaccination in BALB/c mice. BALB/c mice (n = 4) were vaccinated with ChAd63-MVA (A) PfUIS3, (B) PfLSA1 or (C) PfLSAP2, as previously described. Two weeks post-ChAd63 prime and one-week post-MVA boost blood was taken and cellular immunogenicity assessed by ICS, after stimulation for six hours with an overlapping peptide pool to the appropriate antigen. Two weeks post-MVA boost mice were sacrificed, spleens harvested and cellular immunogenicity again assessed by ICS. The proportion of cells at each time-point expressing one, two or three cytokines is shown. The bar chart indicates which cytokines were produced, whilst the slices of the pie chart indicate the proportion of cells producing one (purple), two (orange) or three (black) cytokines.

It was also of interest to assess the polyfunctionality of the induced immune responses in BALB/c mice, given each vaccine provided some degree of protection in this strain. We found for both PfUIS3 and PfLSA1 (Fig 4A and 4B) that approximately one-third of antigen-specific CD8^+^ T cells were dual cytokine producers in the blood after ChAd63 vaccination (primarily IFNγ and TNFα), whilst after the MVA boost this increased to approximately 50% in the blood and 75% in the spleen. For PfLSAP2 vaccination (Fig 4C), the overall response was lower and the majority of antigen-specific CD8^+^ T cells were single cytokine producers (IFNγ or TNFα) following both the prime and the boost.

In previous studies, the contributions of monofunctional or polyfunctional T cells to immunity against malaria has not been clear; polyfunctional cells have been associated with induction of protective immunity after vaccination with sporozoites under chloroquine cover [38, 39], whilst monofunctional CD8^+^ T cells secreting IFNγ have correlated with protective immunity in studies using viral vectors expressing the antigen thrombospondin-related adhesion protein (TRAP) administered with a multiple-epitope string (known as ME-TRAP) [6, 15]. Furthermore, whilst correlates of protection were not identified in the recent irradiated sporozoite vaccination study, the CD8^+^ T cells induced in protected volunteers only secreted IFNγ [40]. Together with our current data, this suggests that different correlates or mechanisms of protection will likely exist depending on the vaccination strategy and antigen/s used. Whilst we were not able to identify specific functional correlates of protection for our vaccines [4], these findings further expand our knowledge of the immunological profile of these vaccines, which may assist in the identification of such correlates in future studies.

## Conclusions

In conclusion, this paper presents a detailed immunological analysis of the protective antigens PfLSA1, PfLSAP2 and PfUIS3. Immunodominant peptides were identified for all antigens delivered in the viral vectored prime-boost regimen, in multiple strains of mice for both PfUIS3 and PfLSAP2 and in BALB/c for PfLSA1. PfLSA1 is not immunogenic in C57BL/6 mice, as previously described [31, 32], and whilst no HLA-A2 responses were identified, responses to this antigen in humans have been previously described [41]. The identification of these immunodominant responses provides a useful tool for subsequent studies on these antigens or vaccines. Furthermore, we clarified the immunological profile of these vaccines in terms of polyfunctionality of CD8^+^ T cells induced in the blood and the spleen.

## Supporting Information

S1 Fig HLA-A2 mice elicit an immune response to the HLA-A2-restricted Influenza A epitope not seen in the background C57BL/6 strain. Female HLA-A2 tg and C57BL/6 mice (n = 4 per strain) were vaccinated intramuscularly with 1x10^6^ pfu MVA expressing the Influenza A nucleoprotein (NP) and matrix protein 1 (MP1) [17] and sacrificed twelve days later. Immune responses to the Influenza A virus were measured by *ex vivo* spleen IFNγ ELISpot. Splenocytes were stimulated with either overlapping peptides to NP+M1 split into three pools (80 peptides total, pool 1 1–26, pool 2 27–52, pool 3 54–80) or the HLA-A2-restricted epitope (located in M1, amino acids 58–66 [18, 19]). Results are expressed as SFU per million splenocytes. Median and individual data points are shown.
(PDF)

S1 Table Primers used to genotype HLA-A2 tg mice. (PDF)

S2 Table *P*. *falciparum* 3D7 UIS3 peptide sequences. (PDF)

S3 Table *P*. *falciparum* 3D7 LSA1 peptide sequences. (PDF)

S4 Table *P*. *falciparum* 3D7 LSAP2 peptide sequences. (PDF)

S5 Table Predicted epitopes within PfUIS3, PfLSA1 and PfLSAP2 using the SYFPEITHI server. (XLSX)

S6 Table Predicted epitopes within PfUIS3, PfLSA1 and PfLSAP2 using the IEDB MHC Class I server. (XLSX)

S7 Table Predicted epitopes within PfUIS3, PfLSA1 and PfLSAP2 using the IEDB MHC Class II server. (XLSX)
