# Supplementary material for: Identification of Immunodominant Responses to the Plasmodium falciparum Antigens PfUIS3, PfLSA1 and PfLSAP2 in Multiple Strains of Mice
Source: PLoS One. 2015 Dec 11;10(12):e0144515. doi: 10.1371/journal.pone.0144515 (PMC4676683; doi:10.1371/journal.pone.0144515)
Supplement: S1 Fig — Female HLA-A2 tg and C57BL/6 mice (n = 4 per strain) were vaccinated intramuscularly with 1x10^6^ pfu MVA expressing the Influenza A nucleoprotein (NP) and matrix protein 1 (MP1) [17] and sacrificed twelve days later. Immune responses to the Influenza A virus were measured by ex vivo spleen IFNγ ELISpot. Splenocytes were stimulated with either overlapping peptides to NP+M1 split into three pools (80 peptides total, pool 1 1–26, pool 2 27–52, pool 3 54–80) or the HLA-A2-restricted epitope (located in M1, amino acids 58–66 [18, 19]). Results are expressed as SFU per million splenocytes. Median and individual data points are shown. (PDF) [file pone.0144515.s001.pdf]

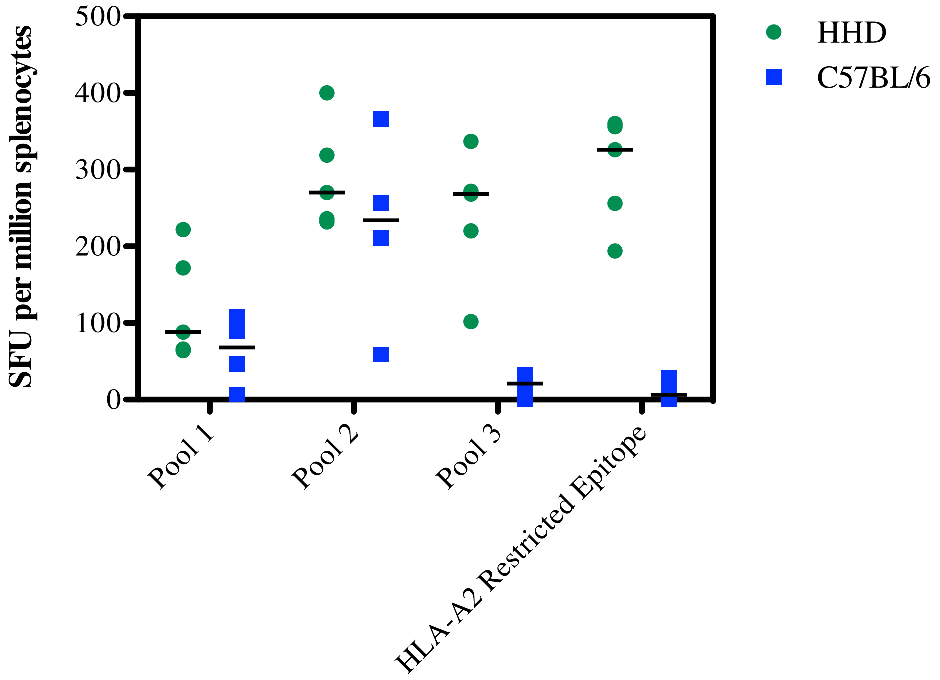

S1 Fig. HLA-A2 tg mice elicit an immune response to the HLA-A2-restricted Influenza A epitope not seen in the background C57BL/6 strain. Female HLA-A2 tg and C57BL/6 mice (n=4 per strain) were vaccinated intramuscularly with $1x10^6$ pfu MVA expressing the Influenza A nucleoprotein (NP) and matrix protein 1 (MP1) (1) and sacrificed twelve days later. Immune responses to the Influenza A virus were measured by *ex vivo* spleen IFNγ ELISpot. Splenocytes were stimulated with either overlapping peptides to NP+M1 split into three pools (80 peptides total, pool 1 1-26, pool 2 27-52, pool 3 54-80) or the HLA-A2-restricted epitope (located in M1, amino acids 58-66 (2, 3)). Results are expressed as SFU per million splenocytes. Median and individual data points are shown.

References

1. Berthoud TK, Hamill M, Lillie PJ, Hwenda L, Collins KA, Ewer KJ, et al. Potent CD8+ T-cell immunogenicity in humans of a novel heterosubtypic influenza A vaccine, MVA-NP+M1. Clinical infectious diseases : an official publication of the Infectious Diseases Society of America. 2011;52(1):1-7. Epub 2010/12/15. doi: 10.1093/cid/ciq015. PubMed PMID: 21148512; PubMed Central PMCID: PMCPMC3060888.

2. Bednarek MA, Sauma SY, Gammon MC, Porter G, Tamhankar S, Williamson AR, et al. The minimum peptide epitope from the influenza virus matrix protein. Extra and intracellular loading of HLA-A2. Journal of immunology (Baltimore, Md : 1950). 1991;147(12):4047-53. Epub 1991/12/15. PubMed PMID: 1721637.

3. Gotch F, Rothbard J, Howland K, Townsend A, McMichael A. Cytotoxic T lymphocytes recognize a fragment of influenza virus matrix protein in association with HLA-A2. Nature. 1987;326(6116):881-2. Epub 1987/04/06. doi: 10.1038/326881a0. PubMed PMID: 2437457.
