# Supplementary material for: Identification of Immunodominant Responses to the Plasmodium falciparum Antigens PfUIS3, PfLSA1 and PfLSAP2 in Multiple Strains of Mice
Source: PLoS One. 2015 Dec 11;10(12):e0144515. doi: 10.1371/journal.pone.0144515 (PMC4676683; doi:10.1371/journal.pone.0144515)
Supplement: S1 Table — (PDF) [file pone.0144515.s002.pdf]

Additional file 1. Primers used to genotype HLA-A2 tg mice.

Gene	Forward primer	Reverse primer	Product Size
H-2^d	GCGGAGAATCCGAGATATGA	CCGCGCTCTGGTTGTAGTAG	157bp
HLA-A2	ACCGTCCAGAGGATGTATGG	CCAGGTAGGCTCTCAACTGC	202bp
Human β2m	TGGCACCTGCTGAGATACTG	CAGTTCCTTTGCCCTCTCTG	713bp
Mouse β2m	CTTGGACCCTTGGTACCTCA	AAGTCCAGTGTTGGGTCAGG	249bp
