# Supplementary material for: Identification of Immunodominant Responses to the Plasmodium falciparum Antigens PfUIS3, PfLSA1 and PfLSAP2 in Multiple Strains of Mice
Source: PLoS One. 2015 Dec 11;10(12):e0144515. doi: 10.1371/journal.pone.0144515 (PMC4676683; doi:10.1371/journal.pone.0144515)
Supplement: S2 Table — (PDF) [file pone.0144515.s003.pdf]

**S2 Table.** ***P. falciparum*** **3D7 UIS3 peptide sequences.**

| Peptide | Sequence |
|---|---|
| UIS3-1 | MKVSKLVLFAHIFFIINILC |
| UIS3-2 | HIFFIINILCQYICLNASKV |
| UIS3-3 | QYICLNASKVNKKGKIAEEK |
| UIS3-4 | NKKGKIAEEKKRKNIKNIDK |
| UIS3-5 | KRKNIKNIDKAIEEHNKRKK |
| UIS3-6 | AIEEHNKRKKLIYYSLIASG |
| UIS3-7 | LIYYSLIASGAIASVAAILG |
| UIS3-8 | AIASVAAILGLGYYGYKKSR |
| UIS3-9 | LGYYGYKKSREDDLYYNKYL |
| UIS3-10 | EDDLYYNKYLEYRNGEYNIK |
| UIS3-11 | EYRNGEYNIKYQDGAIASTS |
| UIS3-12 | YQDGAIASTSEFYIEPEGIN |
| UIS3-13 | EFYIEPEGINKINLNKPIIE |
| UIS3-14 | KINLNKPIIENKNNVDVSIK |
| UIS3-15 | NKNNVDVSIKRYNNFVDIAR |
| UIS3-16 | RYNNFVDIARLSIQKHFEHL |
| UIS3-17 | LSIQKHFEHLSNDQKDSHVN |
| UIS3-18 | SNDQKDSHVNNMEYMQKFVQ |
| UIS3-19 | NMEYMQKFVQGLQENRNISL |
| UIS3-20 | GLQENRNISLSKYQENKAVM |
| UIS3-21 | SKYQENKAVMDLKYHLQKVY |
| UIS3-22 | DLKYHLQKVYANYLSQEEN |
