# Supplementary material for: Identification of Immunodominant Responses to the Plasmodium falciparum Antigens PfUIS3, PfLSA1 and PfLSAP2 in Multiple Strains of Mice
Source: PLoS One. 2015 Dec 11;10(12):e0144515. doi: 10.1371/journal.pone.0144515 (PMC4676683; doi:10.1371/journal.pone.0144515)
Supplement: S3 Table — (PDF) [file pone.0144515.s004.pdf]

S3 Table. ***P. falciparum*** **3D7 LSA1 peptide sequences.**

Peptide	Sequence
LSA1-1	MKHILYISFYFILVNLLIFH
LSA1-2	FILVNLLIFHINGKIIKNSE
LSA1-3	INGKIIKNSEKDEIIKSNLR
LSA1-4	KDEIIKSNLRSGSSNSRNRI
LSA1-5	SGSSNSRNRINEEKHEKKHV
LSA1-6	NEEKHEKKHVLSHNSYEKTK
LSA1-7	LSHNSYEKTKNNENNKFFDK
LSA1-8	NNENNKFFDKDKELTMSNVK
LSA1-9	DKELTMSNVKNVSQTNFKSL
LSA1-10	NVSQTNFKSLLRNLGVSENI
LSA1-11	LRNLGVSENIFLKENKLNKE
LSA1-12	FLKENKLNKEGKLIEHIIND
LSA1-13	GKLIEHIINDDDDKKKYIKG
LSA1-14	DDDKKKYIKGQDENRQEDPQ
LSA1-15	QDENRQEDPQRDLEQRKADT
LSA1-16	RDLEQRKADTKKNLERKKEH
LSA1-17	KKNLERKKEHGDVLAEDLYG
LSA1-18	GDVLAEDLYGRLEIPAIELP
LSA1-19	RLEIPAIELPSENERGYYIP
LSA1-20	SENERGYYIPHQSSLPQDNR
LSA1-21	HQSSLPQDNRGNSRDSKEIS
LSA1-22	GNSRDSKEISIIEKTNRESI
LSA1-23	IIEKTNRESITTNVEGRRDI
LSA1-24	TTNVEGRRDIHKGHLEEKKD
LSA1-25	HKGHLEEKKDGSIKPEQKED
LSA1-26	GSIKPEQKEDKSADIQNHTL
LSA1-27	KSADIQNHTLETVNISDVND
LSA1-28	ETVNISDVNDFQISKYEDEI
LSA1-29	FQISKYEDEISAEYDDSLID
LSA1-30	SAEYDDSLIDEEEDDEDLDE
LSA1-31	EEEDDEDLDEFKPIVQYDNF
LSA1-32	FKPIVQYDNFQDEENIGIYK
LSA1-33	QDEENIGIYKELEDLIEKNE
LSA1-34	ELEDLIEKNENLDDLDEGIE
LSA1-35	NLDDLDEGIEKSSEELSEEK
LSA1-36	KSSEELSEEKIKKGKKYEKT
LSA1-37	IKKGKKYEKTKDNNFKPNDK
LSA1-38	KDNNFKPNDKSLYDEHIKKY
LSA1-39	SLYDEHIKKYKNDKQVNKEK
LSA1-40	KNDKQVNKEKEKFIKSLFHI
LSA1-41	EKFIKSLFHIFDGDNEILQI
LSA1-42	FDGDNEILQIVDELSEDITK
LSA1-43	VDELSEDITKYFMKL
