# Supplementary material for: Identification of Immunodominant Responses to the Plasmodium falciparum Antigens PfUIS3, PfLSA1 and PfLSAP2 in Multiple Strains of Mice
Source: PLoS One. 2015 Dec 11;10(12):e0144515. doi: 10.1371/journal.pone.0144515 (PMC4676683; doi:10.1371/journal.pone.0144515)
Supplement: S4 Table — (PDF) [file pone.0144515.s005.pdf]

S4 Table. ***P. falciparum*** **3D7 LSAP2 peptide sequences.**

Peptide	Sequence
LSAP2-1	MKRGLCCVLLLCGAVFVSPS
LSAP2-2	LCGAVFVSPSQEIHARFRRG
LSAP2-3	QEIHARFRRGMWLCKRGLSV
LSAP2-4	MWLCKRGLSVNDTTKCDVPC
LSAP2-5	NDTTKCDVPCKDFYMLFLSN
LSAP2-6	KDFYMLFLSNKKEKIKCGTF
LSAP2-7	KKEKIKCGTFFGYIFLSKFM
LSAP2-8	FGYIFLSKFMKLSISLLLLA
LSAP2-9	KLSISLLLLALIQNILLSNV
LSAP2-10	LIQNILLSNVSLISGSHLYK
LSAP2-11	SLISGSHLYKRNSRKFAEGY
LSAP2-12	RNSRKFAEGYMKGSGSEKNV
LSAP2-13	MKGSGSEKNVYLSNKNKEIN
LSAP2-14	YLSNKNKEINMNQQSDNKMC
LSAP2-15	MNQQSDNKMCDECDDMNQPG
LSAP2-16	DECDDMNQPGDVNKNDKTSN
LSAP2-17	DVNKNDKTSNDQANSSDSDC
LSAP2-18	DQANSSDSDCEPLPFGLKPS
LSAP2-19	EPLPFGLKPSDLNRKVTEED
LSAP2-20	DLNRKVTEEDLERMIIELPG
LSAP2-21	LERMIIELPGKLERKDMYLI
LSAP2-22	KLERKDMYLIWHYSHSLLRD
LSAP2-23	WHYSHSLLRDKFNKMKSSLW
LSAP2-24	KFNKMKSSLWSICGKLAHEH
LSAP2-25	SICGKLAHEHKLPFKIKMKK
LSAP2-26	KLPFKIKMKKWWKCCGHVTD
LSAP2-27	WWKCCGHVTDELLIKEHDDY
LSAP2-28	ELLIKEHDDYNSIYNYINNE
LSAP2-29	NSIYNYINNESSSREQFLIF
LSAP2-30	SSSREQFLIFLNMIKHSWTT
LSAP2-31	LNMIKHSWTTFTMETFIKCK
LSAP2-32	FTMETFIKCKISLENNMRNV
LSAP2-33	ISLENNMRNVTN
